# The interplay between NCAM and FGFR signalling underlies ovarian cancer progression

**DOI:** 10.3332/ecancer.2011.226

**Published:** 2011-10-20

**Authors:** N Colombo, U Cavallaro

**Affiliations:** Department of Gynaecological Oncology (NC), and Molecular Medicine Program (UC), European Institute of Oncology, Milano, Italy

## Abstract

Epithelial ovarian carcinoma (EOC) is an aggressive neoplasm, which has often disseminated to peritoneal cavity at the time of diagnosis. In order to better understand the molecular mechanisms behind EOC progression, we investigated the expression and functional role of neural cell adhesion molecule (NCAM) in this tumour type.

While NCAM is absent from normal ovarian surface epithelium, it is highly expressed in a subset of EOC, and its expression is associated with high tumour grade.

In a recent publication [1], we have demonstrated that NCAM stimulates EOC cell migration and invasion *in vitro* and promotes metastatic dissemination in mice. This activity is mediated by its interaction with the fibroblast growth factor receptor (FGFR). Further, interfering with the NCAM/FGFR interplay in mice resulted in the inhibition of the metastatic dissemination of EOC, suggesting that this molecular interaction may represent a valuable therapeutic target.

Epithelial ovarian cancer (EOC) is a relatively common disease and the most deadly of all gynaecological tumours. Although survival rates have improved in the last 30 years, its prognosis is still poor when compared to many other common neoplasms, with 5-year survival rates of only about 40%. There are two main reasons for this. The early symptoms of EOC are vague and mimic conditions that are much more common and less severe, which has led it to be termed ‘the silent killer’, and it is aggressive, spreading rapidly from the ovary into the other organs in the peritoneal cavity. Most cases are therefore only diagnosed when the cancer has already spread and when even a combination of surgery and chemotherapy rarely lead to a complete cure.

It is clear that there is a pressing need for drugs that target the specific molecular mechanisms involved in the development of epithelial ovarian cancer. However, our knowledge of this, and of the genetic lesions that underlie them, is still incomplete. In a recent publication [1], we were able to identify one potential mechanism, the interplay between an adhesion molecule and a growth factor, underlying ovarian cancer development. We suggest that this interplay may represent a potential novel drug development target.

Most experts think that epithelial ovarian cancer develops from a single layer of epithelial cells lining the ovary known as the ovarian surface epithelium. When this tumour metastasizes, it does so, unlike other cancers, by shedding small clusters of cells that diffuse through the peritoneal cavity and adhere to the organs within. This process implies the involvement of proteins known as adhesion molecules. Neural cell adhesion molecule (NCAM) is a cell surface glycoprotein that was, as its name implies, first characterized as regulating neural migration and intercellular adhesion in the nervous system. It is, however, known to have a much wider spectrum of activities, and to be frequently expressed in EOC and other cancers. NCAM has been proposed to regulate the function of members of the fibroblast growth factor receptor (FGFR) family. These growth factor receptors are tyrosine kinases that trigger signalling cascades when bound by growth factors and other molecules, and aberrant FGFR signalling has been implicated in the progression of many cancers, including epithelial ovarian cancer.

Following a series of experiments described in more detail elsewhere (2,3), we demonstrated that NCAM interacts with FGFR and, based on the importance of both adhesion molecules and growth factor receptor signalling in ovarian cancer development, set out to test the theory that NCAM binding can trigger FGFR signalling and thus ovarian cancer development. First, we tested normal ovarian tissue, precancerous lesions, and primary and metastatic EOC for expression of NCAM using immunohistochemical staining. No expression was found in any of the normal or precancerous tissue; in contrast, a number of the cancer samples tested positive, with a higher percentage of metastatic than of primary lesions showing NCAM expression. Furthermore, expression was observed to be particularly strong at the edges of invasive tumours, adding credence to the hypothesis that this molecule is involved in promoting cancer invasion and metastasis. Most of the primary EOC samples were shown by the same method to express fibroblast growth factor receptors, particularly expression of FGFR1, FGFR3 and FGFR4, and NCAM was shown to correlate with that of the FGFR genes.

Subsequently, we investigated the role of the interplay between NCAM and FGFR signalling using tumour tissue taken from a transgenic mouse model of ovarian cancer that expresses a high level of NCAM at the tumour boundary. Expression of the adhesion molecule was knocked down using a short hairpin RNA, which had no effect on the proliferation of the cancer cells but significantly reduced their ability to migrate. Whether this migration was dependent on NCAM-FGFR interplay, however, had not yet been shown. Reducing FGFR signalling with a selective small-molecule inhibitor, PD173074 did not reduce migration any further, which suggested that these molecules act in the same pathway.

Having shown an interaction between NCAM and FGFR to be necessary for ovarian cancer cell migration, we set out to investigate whether it was sufficient using NCAM-negative human ovarian cancer cell lines. The forced expression of NCAM in these cells transformed relatively indolent cancer cells into invasive ones. However, this increase in invasion did not occur when the cells were transfected instead with an engineered form of NCAM lacking the domain involved in FGFR binding. As this domain deletion did not affect the protein's level, folding, or location at the cell surface, this implied that the FGFR-NCAM interaction was implicated in cell migration. Further evidence from this came from the fact that Encamin-C, a peptide derived from NCAM that recapitulates FGFR binding and activation, could both stimulate growth factor activity and promote cell migration.

The next step was to determine the extent to which NCAM-FGFR signalling can be implicated in tumour invasion *in vivo*. To do this, we assessed the invasive potential in a mouse model of xeno-transplanted human ovarian cancer cells with no endogenous NCAM expression. Injection of these tumours with wild-type NCAM, but not with the NCAM mutant lacking the FGFR interaction domain, transformed smooth, noninvasive tumours into much more aggressive ones that infiltrated the peritoneal cavity. The same tumour cells engineered to express either wild-type or mutant NCAM were then injected into the peritoneal cavities of immunodeficient mice; after 7 weeks, only the cells expressing the wild-type NCAM had formed tumours that had metastasized to the omentum (Figure 1), bowel, liver and diaphragm. These peritoneal organs are the most common sites of metastases in human ovarian cancer.

If this interaction is to provide a novel target for drugs against ovarian cancer, it must be possible to interfere with it using either an antibody or a small-molecule inhibitor. This question was addressed by injecting mice bearing ovarian tumour xenografts that expressed wild-type NCAM with either an antibody known to target the NCAM-FGFR interface or a control antibody. A significant decrease in peritoneal metastases was observed only in mice injected with the NCAM-FGFR-targeted antibody.

In conclusion, several lines of evidence taken from our *in vitro* and *in vivo* studies implicate the specific interaction between the adhesion molecule NCAM and members of the fibroblast growth factor receptor family in the invasive phenotype that is common in epithelial ovarian cancer, and thus in cancer progression. Furthermore, the fact that this interaction can be blocked by a targeted monoclonal antibody provides an important proof of principle of this interaction as a novel therapeutic target. An antibody or small-molecule inhibitor of this novel protein–protein interaction would represent an important addition to the armoury of the clinicians targeting such a devastating disease.

## Figures and Tables

**Figure 1:**
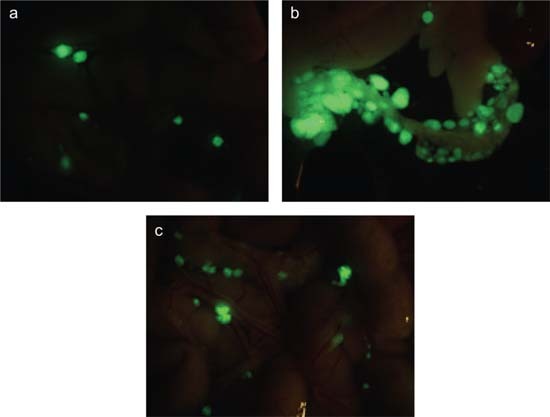
Immunodeficient mice were subjected to intra-peritoneal injection of SKOV3 ovarian carcinoma cells co-transfected with GFP and with either (a) an empty vector, (b) wild-type NCAM or (c) a deletion mutant of NCAM lacking the FGFR-binding domain. Pictures show masses o GFP-positive tumour cells in the omental region. NCAM-expressing ovarian cancer cells exhibit a higher rate of peritoneal dissemination (b) as compared to mock-transfected cells (a). This NCAM-dependent invasive phenotype requires the interaction with FGFR (c).
